# Generalized and Improved ***(G′/G)***-Expansion Method for (3+1)-Dimensional Modified KdV-Zakharov-Kuznetsev Equation

**DOI:** 10.1371/journal.pone.0064618

**Published:** 2013-05-31

**Authors:** Hasibun Naher, Farah Aini Abdullah, M. Ali Akbar

**Affiliations:** 1 School of Mathematical Sciences, Universiti Sains Malaysia, Penang, Malaysia; 2 Department of Mathematics and Natural Sciences, BRAC University, Dhaka, Bangladesh; 3 Department of Applied Mathematics, University of Rajshahi, Rajshahi, Bangladesh; University of Catania, Italy

## Abstract

The generalized and improved 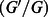 **-**expansion method is a powerful and advantageous mathematical tool for establishing abundant new traveling wave solutions of nonlinear partial differential equations. In this article, we investigate the higher dimensional nonlinear evolution equation, namely, the (3+1)-dimensional modified KdV-Zakharov-Kuznetsev equation via this powerful method. The solutions are found in hyperbolic, trigonometric and rational function form involving more parameters and some of our constructed solutions are identical with results obtained by other authors if certain parameters take special values and some are new. The numerical results described in the figures were obtained with the aid of commercial software Maple.

## Introduction

Nonlinear partial differential equations (PDEs) are widely used to describe complex physical phenomena in different branches of mathematical physics, engineering sciences and other technical arenas. The analytical solutions of nonlinear evolution equations (NLEEs) have now become a more exciting topic for a diverse group of scientists. In recent years, they established several powerful methods to obtain exact solutions. For example, the Backlund transformation method [1], [2], the inverse scattering method [3], the truncated Painleve expansion method [4], the Weierstrass elliptic function method [5], the Hirota’s bilinear transformation method [6], the Jacobi elliptic function expansion method [7]–[9], the generalized Riccati equation method [10], the tanh-coth method [11]–[13], the F-expansion method [14], [15], the direct algebraic method [16], the Exp-function method [17]–[23] and others [24]–[28].

Every method has some restrictions in their implementations. Basically, there is no integrated method which could be utilized to handle all types of nonlinear PDEs. Another powerful and effective method has been presented by Wang *et al.* [29] to construct exact traveling wave solutions and called the 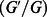 **-**expansion method. In this method, they employed the second order linear ordinary differential equation (ODE) 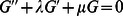 where 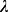 and 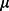 are arbitrary constants. Afterwards, several researchers applied this basic method to obtain traveling wave solutions for different nonlinear PDEs [30]–[33]. Recently, Zhang *et al.* [34] extended the 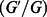 **-**expansion method which is called the improved 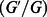 **-**expansion method. In this method, 
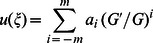
is used as traveling wave solutions, where either 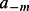 or 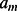 may be zero, but both 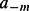 and 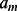
cannot together be zero. And a diverse group of scientists implemented this method to establish new traveling wave solutions of NLEEs [35]–[38].

Very recently, Akbar *et al*. [39] extended and improved this method by using 
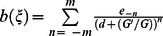
 as traveling wave solutions, where either 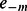 or 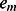 may be zero, but both 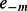 and 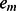 cannot be zero together and is called the generalized and improved 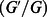 **-**expansion method. This method could be applied for generating a rich class of traveling wave solutions because, in this method, an additional variable 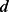 is applied and it can produce more general and abundant solutions. If 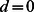, we can obtain the same solutions according to Zhang *et al.* [34].

The aim of this work is that, we concentrate to find more general and abundant traveling wave solutions of the (3+1)-dimensional modified KdV-Zakharov-Kuznetsev equation by implementing the generalized and improved 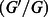 **-**expansion method.

## Description of the Method

Let us consider a general nonlinear PDE:
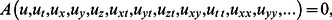
(1)where 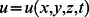 is an unknown function, 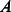 is a polynomial in its arguments and the subscripts stand for the partial derivatives.

The main steps of the method [39] are as follows:

### 

#### Step 1

We suppose the traveling wave variable:
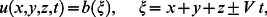
(2)where 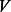 is the speed of the traveling wave. Using Eq. (2), Eq. (1) is converted into an ordinary differential equation for 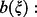


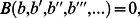
(3)where the superscripts indicate the ordinary derivatives with respect to 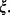


#### Step 2

According to possibility, Eq. (3) can be integrated term by term one or more times, yielding constant(s) of integration. The integral constant may be zero, for simplicity.

#### Step 3

Suppose that the traveling wave solution of Eq. (3) can be expressed by a polynomial in 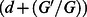 as follows:
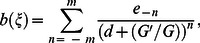
(4)where either 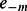 or 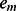 may be zero, but both 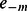 and 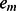 cannot be zero simultaneously, 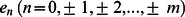 and 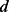 are arbitrary constants to be determined later, and 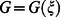
satisfies the following second order linear ODE:
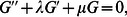
(5)where 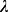 and 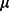 are constants.

#### Step 4

To determine the positive integer 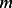, taking the homogeneous balance between the highest order nonlinear terms and the highest order derivatives appearing in Eq. (3). If the degree of 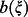 is 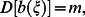 then the degree of the other expression would be as follows:
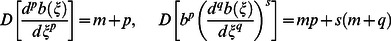
(6)


#### Step 5

Substituting Eq. (4) and Eq. (5) into Eq. (3) together with the value of 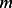 obtained in Step 4 yields polynomials in 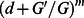 and 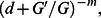 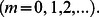 Collecting each coefficient of the resulted polynomials to zero, we obtain a set of algebraic equations for 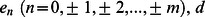 and 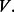


#### Step 6

Suppose that the value of the constants 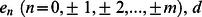 and 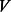
can be found by solving the algebraic equations which are obtained in step 5. Since the general solution of Eq. (5) is well known to us, substituting the values of 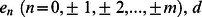 and 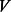 into Eq. (4), we can obtain more general type and new exact traveling wave solutions of the nonlinear partial differential equation (1).

## Application of the Method

In this section, we apply the generalized and improved 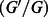 **-**expansion method to establish more general and some new exact traveling wave solutions of the well known (3+1)-dimensional modified KdV-Zakharov-Kuznetsev equation.

Let us consider the (3+1)-dimensional modified KdV-Zakharov-Kuznetsev equation followed by Zayed [40]:
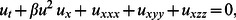
(7)


Now, we use the wave transformation Eq. (2) into the Eq. (7), which yields:
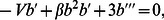
(8)


Eq. (8) is integrable, therefore, integrating with respect 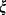 once yields:
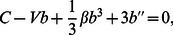
(9)where 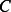 is an integral constant which is to be determined later.

Taking the homogeneous balance between 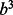
and 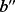 in Eq. (9), we obtain 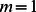.

Therefore, the solution of Eq. (9) is of the form:
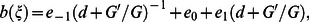
(10)where 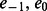 and 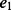 are constants to be determined.

Substituting Eq. (10) together with Eq. (5) into Eq. (9), the left-hand side is converted into polynomials in 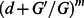 and 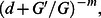 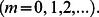 We collect each coefficient of these resulting polynomials to zero, yielding a set of simultaneous algebraic equations (for simplicity, which are not presented) for 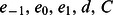 and 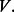 Solving these algebraic equations with the help of symbolic computation system Maple 13, we obtain following.

**Case 1:**

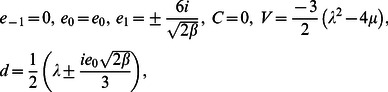
(11)where 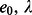 and 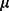 are free parameters.

**Case 2:**

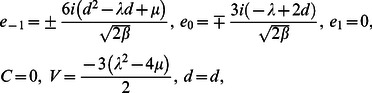
(12)where 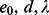 and 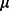 are free parameters.

**Case 3:**

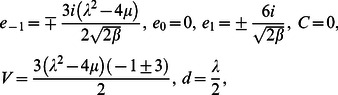
(13)where 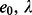 and 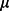 are free parameters.

**Case 4:**

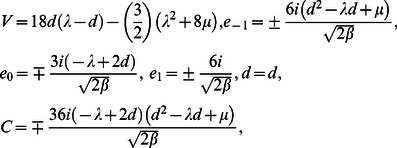
(14)where 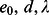 and 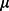 are free parameters.

Substituting the general solution Eq. (5) into Eq. (10), we obtain the following.

When 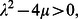 we obtain following hyperbolic function solution:
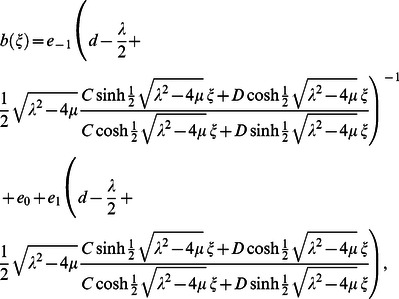
(15)where 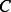 and 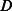 are arbitrary constants, if 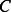 and 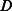 take particular values, various known solutions can be rediscovered.

When 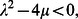 we obtain the trigonometric function solution:
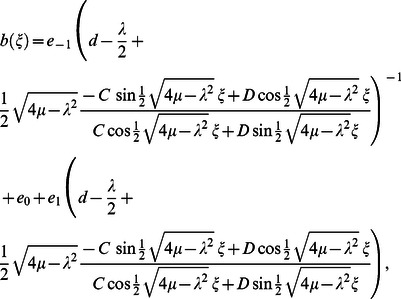
(16)where 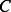 and 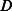 are arbitrary constants, if 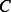 and 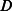 take particular values, various known solutions can be rediscovered.

When 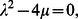 we obtain the rational function solution:
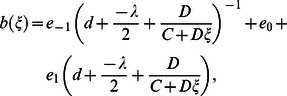
(17)


For case 1, substituting Eq. (11) into Eq. (15) and simplifying, yields following traveling wave solutions when 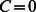 but 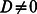 and 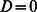 but 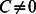) respectively:
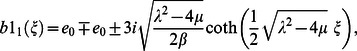


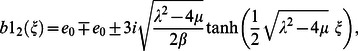



Again, substituting Eq. (11) into Eq. (16) and simplifying, our exact solutions become when 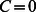 but 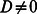 and 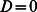 but 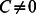 respectively:
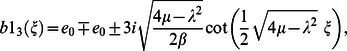


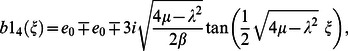



Moreover, substituting Eq. (11) into Eq. (17) and simplifying, our obtained solutions becomes:
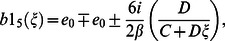
where 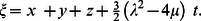


Similarly, for case 2, substituting Eq. (12) into Eq. (15) and simplifying, yields following traveling wave solutions when 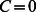 but 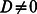; 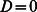 but 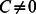 respectively:
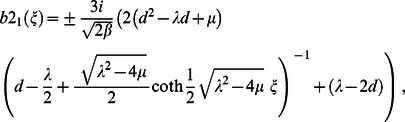


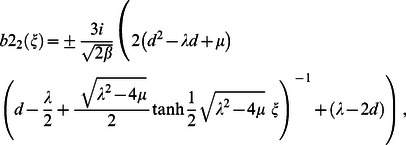



Substituting Eq. (12) into Eq. (16) and simplifying, yields following traveling wave solutions when 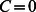 but 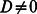; 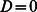 but 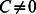 respectively:
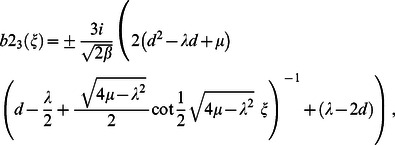


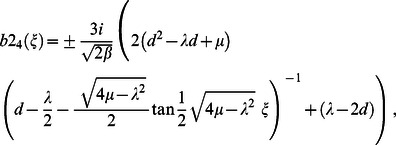



Finally, substituting Eq. (12) into Eq. (17) and simplifying, yields following traveling wave solutions:
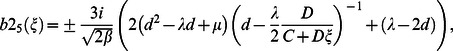
where 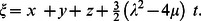


Again, for case 3, substituting Eq. (13) into Eq. (15) and simplifying, yields following traveling wave solutions when 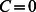 but 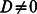; 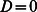 but 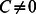 respectively:
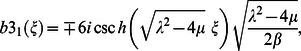



Substituting Eq. (13) into Eq. (16) and simplifying, yields following traveling wave solutions when 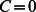 but 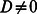; 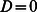 but 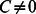 respectively:
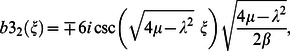



Substituting Eq. (13) into Eq. (17) and simplifying, yields following traveling wave solutions:
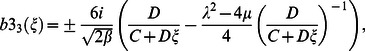
where 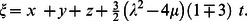


Moreover, for case 4, substituting Eq. (14) into Eq. (15) and simplifying, yields following traveling wave solutions (if 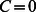 but 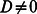; 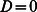 but 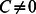) respectively:


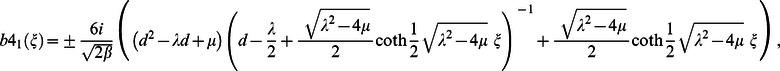
 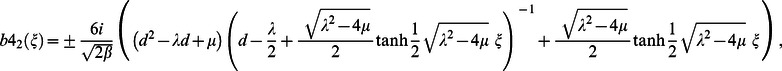 for case 4, substituting Eq. (14) into Eq. (16) and simplifying, yields following traveling wave solutions when 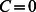 but 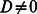; 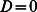 but 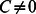 respectively:


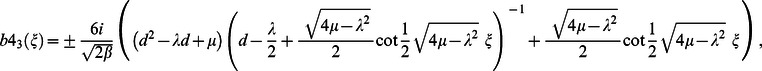
 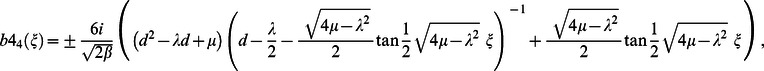 for case 4, substituting Eq. (14) into Eq. (17) and simplifying, yields following traveling wave solutions:
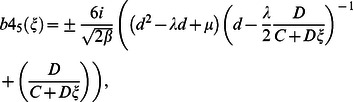
where 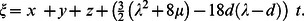


## Results and Discussion

**Figure 1:**
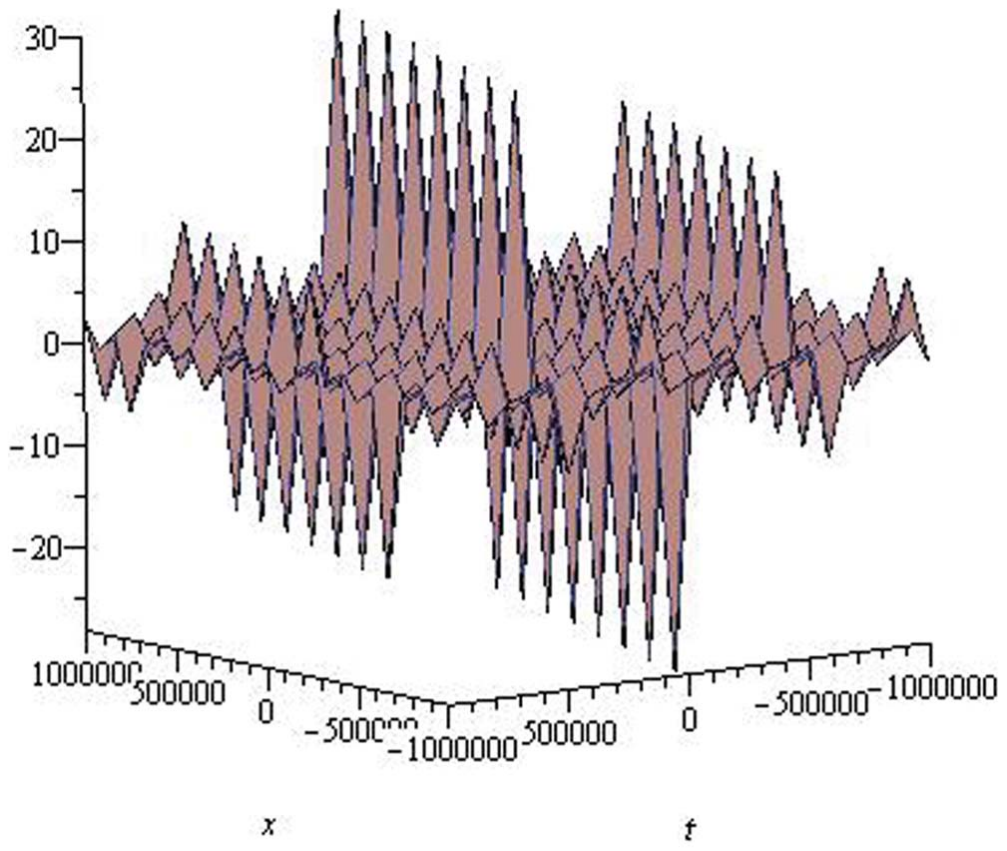
Solitons solution for 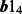. 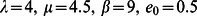
.

**Figure 2:**
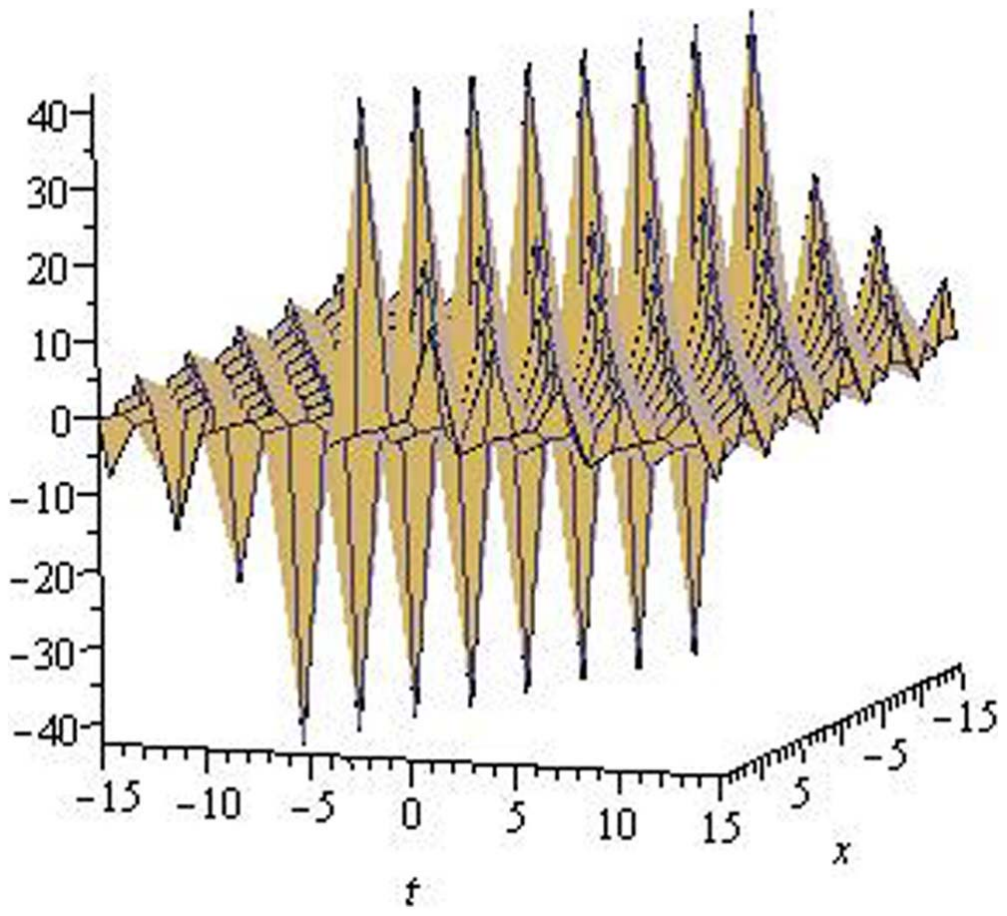
Solitons solution for 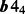. 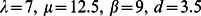
.

**Figure 3:**
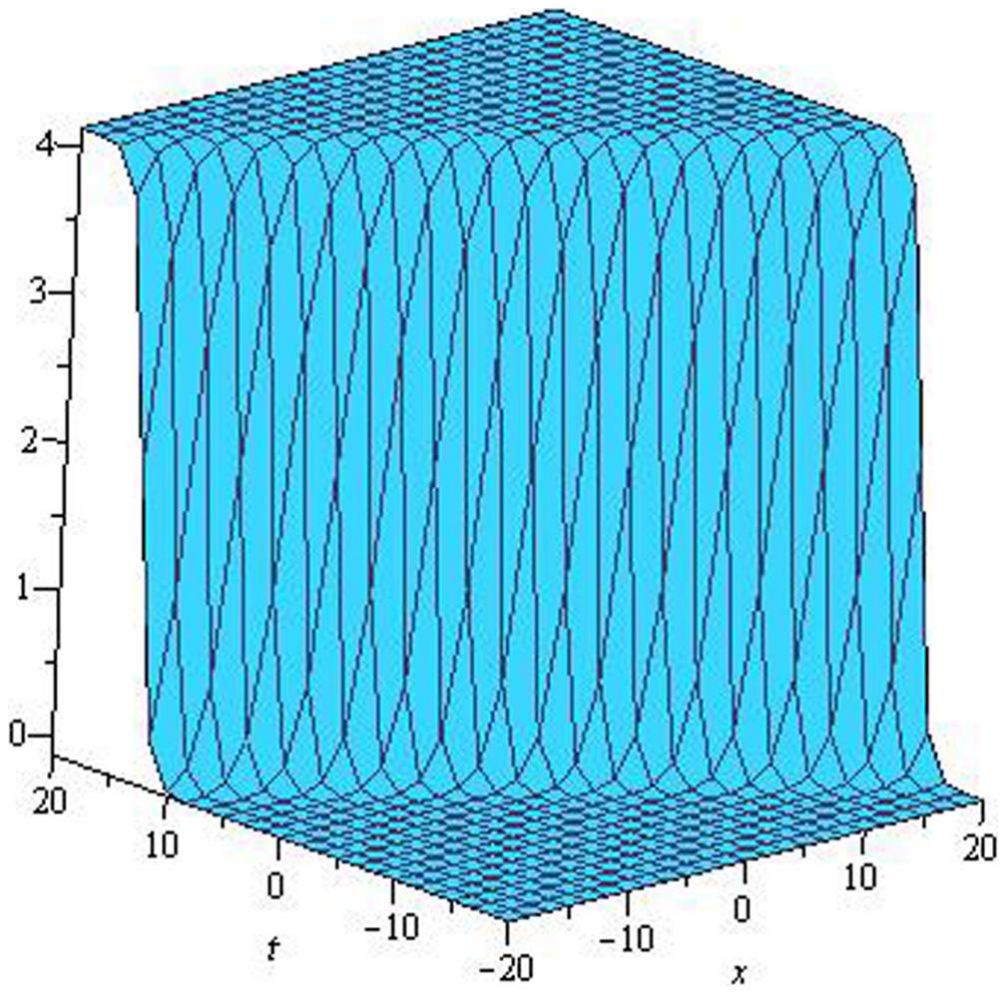
Periodic solution for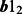. 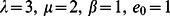
.

**Figure 4:**
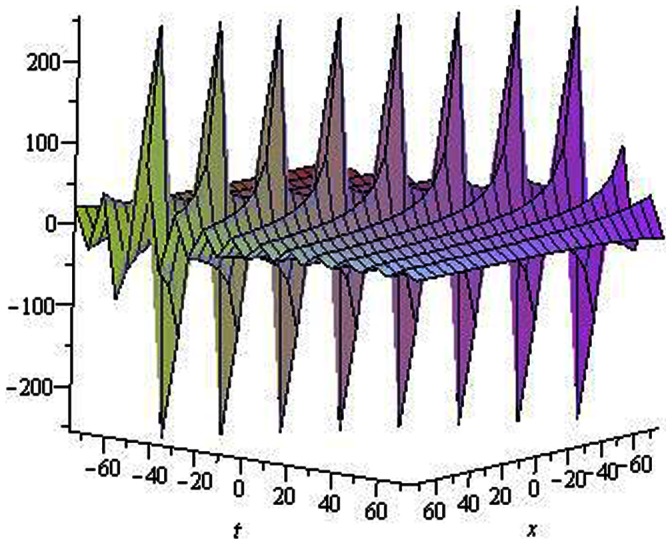
Solitons solution for 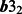. **
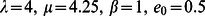
**.

**Figure 5:**
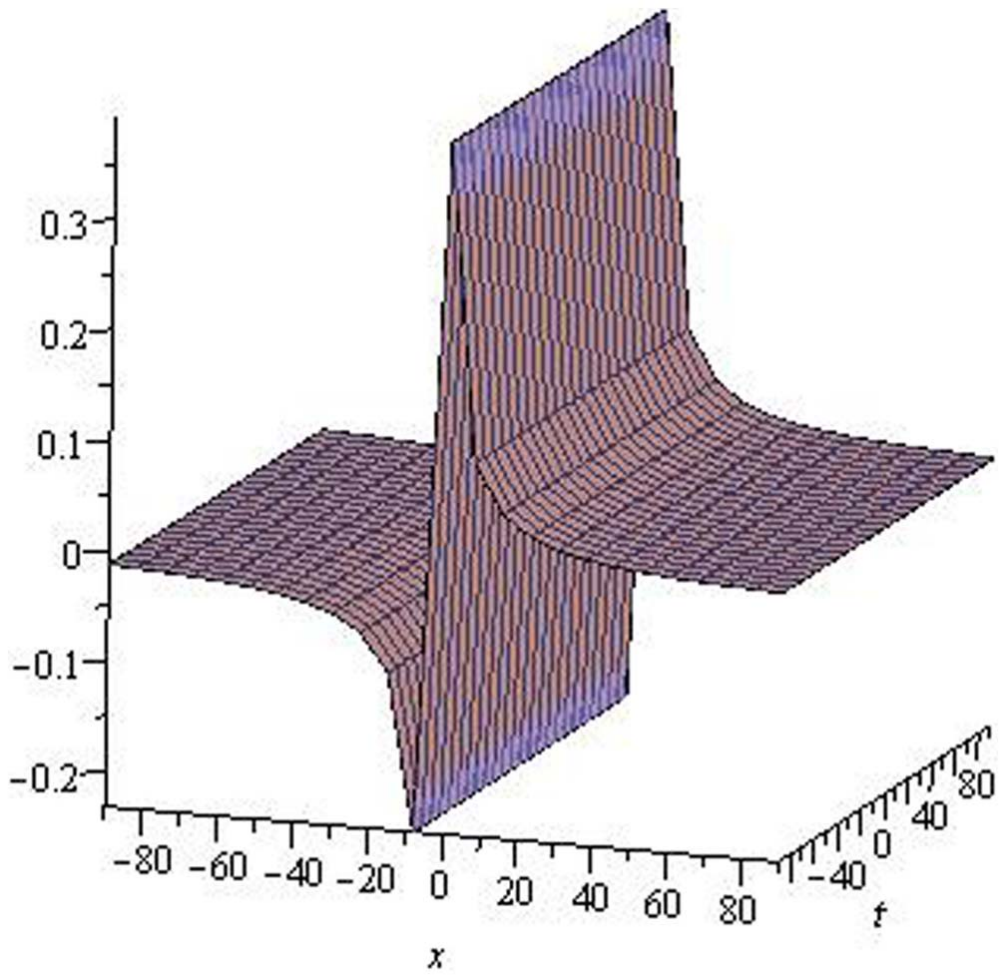
Periodic solution for 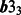. **
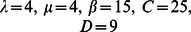
**.

**Figure 6:**
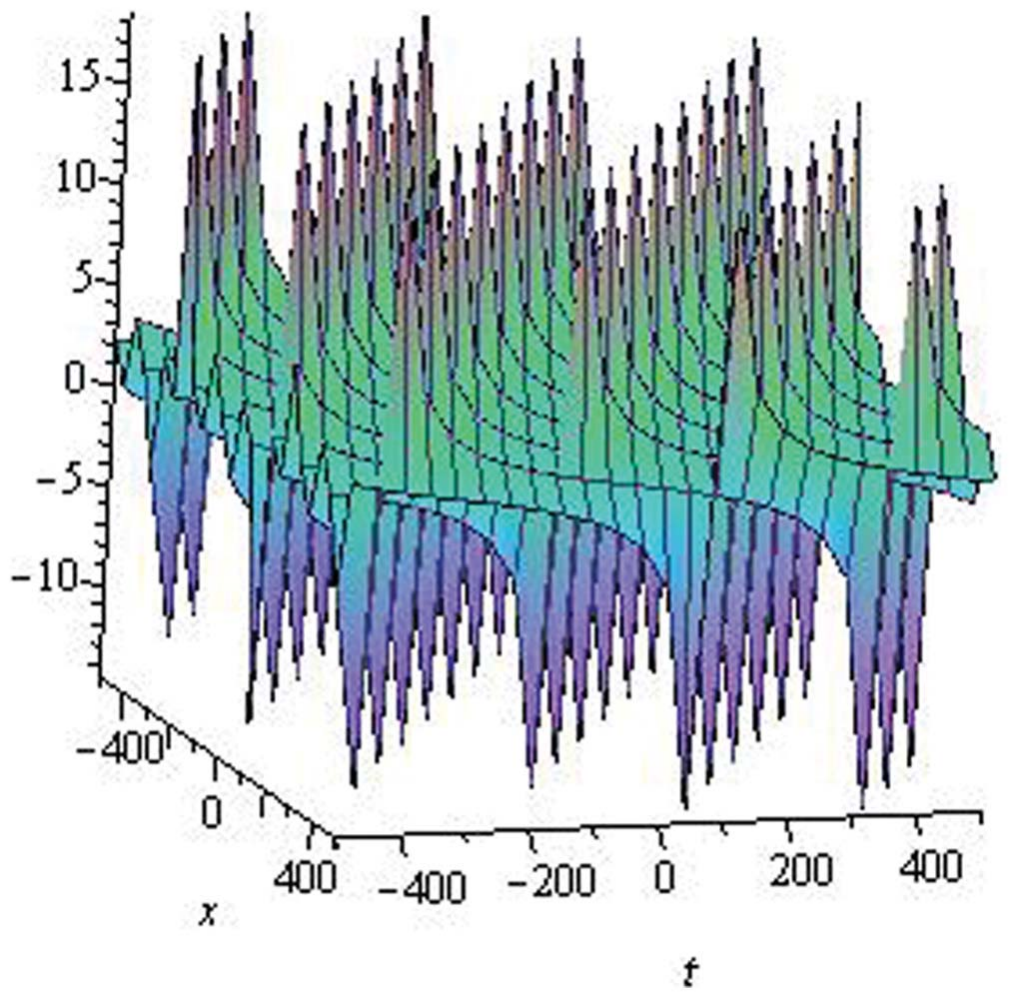
Solitons solution for 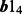. 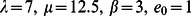
.

**Figure 7:**
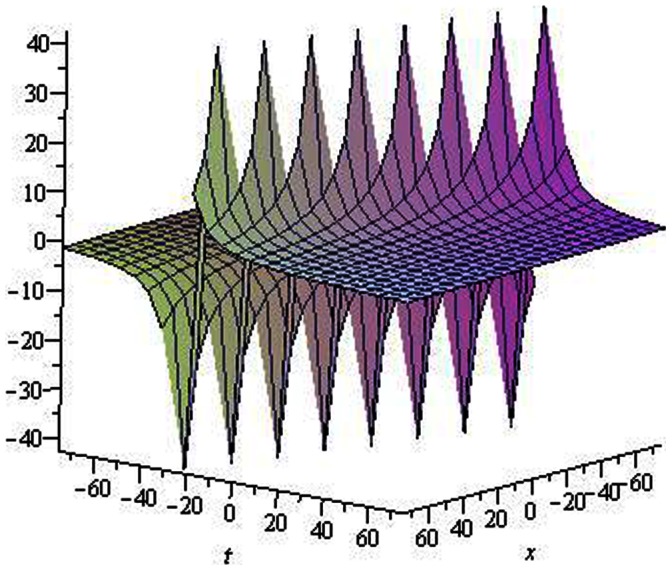
Solitons solution for 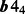. 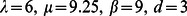
.

**Figure 8:**
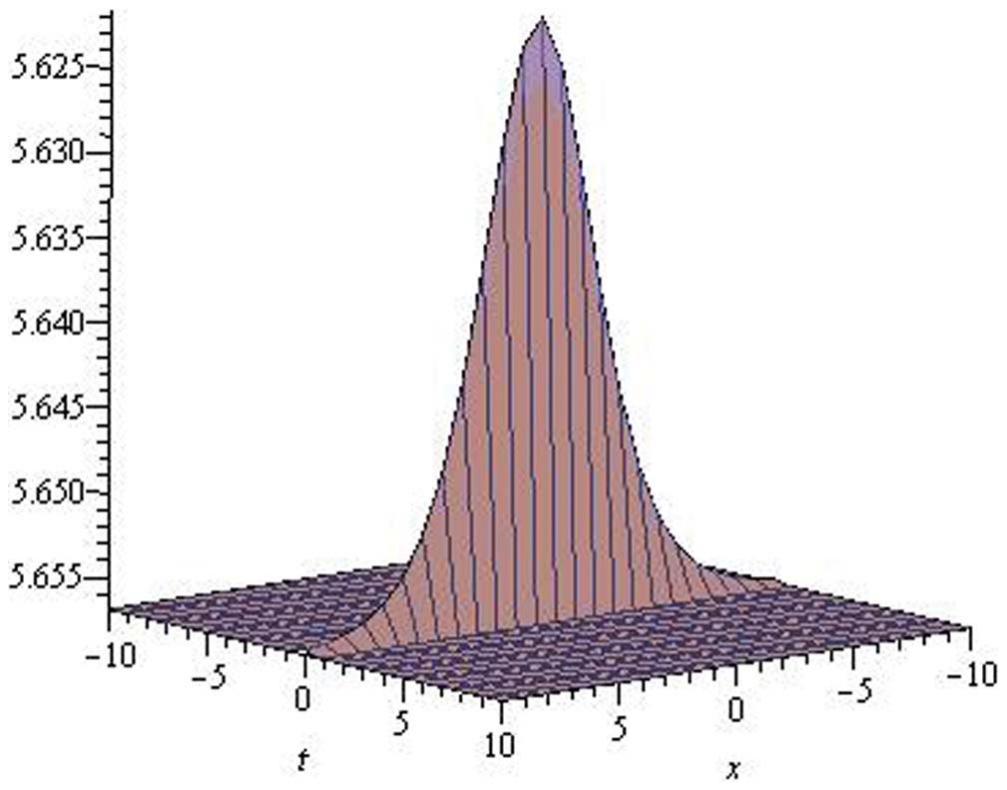
Solitons solution for 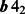. 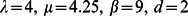
.

The higher dimensional modified KdV-Zakharov-Kuznetsev equation has been solved by many authors by implementing different methods. For example, Zayed [40] executed the basic 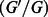-expansion method, Xu [41] used the elliptic equation method, Naher *et al.* [18] applied the Exp-function method furthermore, they [42] employed the improved 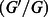-expansion method to obtain traveling wave solutions of this mentioned equation. But in this article, we construct more general and new exact traveling wave solutions by applying the generalized and improved 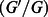-expansion method with an additional free parameter 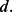 The obtained solutions would be useful to understand the mechanism of the complicated nonlinear physical phenomena in a wave interaction. Moreover, some solutions are identical with already published results which are described in table 1. Beyond this table, we obtain new exact solutions 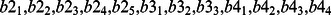 and 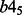
which are not established in the previous literature. Also, solutions 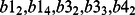
and 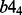
are depicted in Figures 1, 2, 3, 4, 5, 6, 7, 8.

**Table 1:** Comparison between Naher *et al.* [42] solutions and Newly obtained solutions.

Naher *et al.* [42]	New solutions
i.If 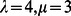 and 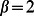 solution Eq. (15) becomes: 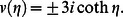	i. If 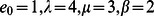 and 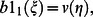 solution 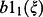 becomes: 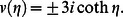
ii. If 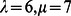 and 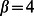 solution Eq. (16) becomes: 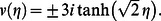	ii. If 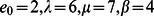 and 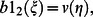 solution 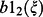 becomes: 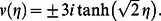
iii. If 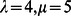 and 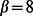 solution Eq. (19) becomes: 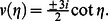	iii. If 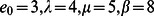 and 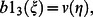 solution 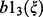 becomes: 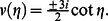
iv. If 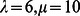 and 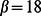 solution Eq. (20) becomes: 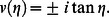	iv. If 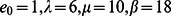 and 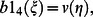 solution 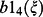 becomes: 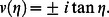
v. If 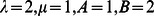 and 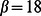 solution Eq. (22) becomes: 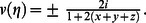	v. If 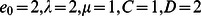 and 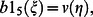 solution 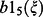 becomes: 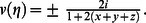

### Graphical Presentations of Some Solutions

The graphical presentations of some solutions are illustrated in Figures 1, 2, 3, 4, 5, 6, 7, 8 with the aid of commercial software Maple.

### Conclusions

In this article, the generalized and improved 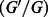 **-**expansion method is implemented to produce plentiful new traveling wave solutions of the (3+1)-dimensional modified KdV-Zakharov-Kuznetsev equation. The used method has many advantages: it is straightforward and concise. Further, the obtained solutions reveal that this method is a promising mathematical tool because it can furnish a different class of new traveling wave solutions with free parameter of distinct physical structures. Subsequently, this prominent method could be more effectively used to solve various nonlinear partial differential equations which regularly arise in science, engineering and other technical arenas.
